# Evaluation of Changes in Condylar Cartilage Thickness Using MRI and Ultrasound Imaging in Patients Treated by Mandibular Advancement With Myofunctional Appliance: An In-Vivo Pilot Study

**DOI:** 10.7759/cureus.16338

**Published:** 2021-07-12

**Authors:** Sonali Deshmukh, Sachin Durkar, Amit Kharat, Suramya Shiranjani, Pranav Ajmera

**Affiliations:** 1 Orthodontics and Dentofacial Orthopaedics, Dr. D.Y. Patil Dental College, Dr. D.Y. Patil Vidyapeeth (DPU), Pune, IND; 2 Orthodontics, Dr. D.Y. Patil Dental College, Dr. D.Y. Patil Vidyapeeth (DPU), Pune, IND; 3 Musculoskeletal Radiology, Dr. D.Y. Patil Medical College, Hospital and Research Center, Dr. D.Y. Patil Vidyapeeth (DPU), Pune, IND; 4 Dentistry, Dr. D.Y. Patil Dental College, Dr. D.Y. Patil Vidyapeeth (DPU), Pune, IND; 5 Radiology, Dr. D.Y. Patil Medical College, Hospital and Research Center, Dr. D.Y. Patil Vidyapeeth (DPU), Pune, IND

**Keywords:** condylar cartilage, mri, usg, myofunctional appliance therapy, functional therapy

## Abstract

Introduction

The changes occurring due to growth modulation of the condylar cartilage act as an important mechanism for mandibular advancement using myofunctional appliance therapy. So this study aims to evaluate the condylar cartilage thickness by using MRI and USG in patients undergoing myofunctional appliance therapy for mandibular advancement with the null hypothesis being that there are no changes seen in the thickness of condylar cartilage in growing children.

Materials and methods

A prospective evaluation of samples having skeletal Class-II malocclusion ranging between cervical vertebral maturation index (CVMI) stage II and III, requiring twin block functional therapy was performed. Ten patients were selected randomly who underwent MRI and USG in the open and close positions for the evaluation of condylar cartilage thickness and the dimensional changes in the width of the right and left condyle in mm at T0 and T1.

Result

There was no statistically significant difference between the values interpreted by MRI or USG imaging when compared at T0 and T1 and in the open and closed mouth on the left and right sides. At T0, the mean thickness noted was 0.49 mm and 0.48 mm during opening and closing on the left side and 0.52 mm in both positions on the right side. At T1, the mean thickness noted was 0.8 and 0.79mm during opening and closing on the left side, whereas it was 0.81 mm in both positions on the right side.

Conclusion

The condylar cartilage thickness increases significantly after twin block therapy suggestive of mandibular growth in skeletal class II malocclusion. It can be inferred that both MRI and USG carry equal diagnostic interpretation, as there was no statistically significant difference between the two imaging modalities.

## Introduction

The prevalence of class-II malocclusion is about 8.37% in the Indian population. Class-II division 1 malocclusion is often associated with discrepancies like narrow maxilla, high arched palate, increased overjet, and microgenia. However, the most commonly associated feature is mandibular skeletal retrusion. Functional appliance therapy serves to enhance mandibular growth, thereby correcting the skeletal discrepancy [1].

Most of the functional appliances are designed for propagating forward growth of the mandible and inducing displacement of the mandibular condyles in a downward and forward direction. Adaptive remodeling occurs on both the articular surfaces of the temporomandibular joint, thrusting the mandible forward and improving the sagittal position of the mandible relative to the maxilla [1].

Mandibular advancement induced by functional therapy for skeletal Class-II malocclusion is a principal factor that directs cellular activities during tissue morphogenesis and mechanical stress. Forward positioning of the mandible is a primary event that is followed by adaptive remodeling in the mandibular condylar cartilage (MCC) and the glenoid fossa (GF). Bone remodeling occurs by the expression of endogenous regulatory factors of cells in the mandibular condyle through an endochondral and intramembranous ossification in the GF [2].

As conventional imaging systems acquire several limitations, a detailed study of the temporomandibular joint (TMJ) structure using magnetic resonance imaging (MRI) and ultrasound imaging is substantiated since these imaging modalities offer a method for diagnostic superiority for the evaluation of both the soft and hard tissues of the TMJ. MRI facilitates the non-invasive assessment of various tissues by providing high-resolution images that account for apparent differentiation among soft tissues. The clinical application of MRI has prompted a better understanding of the anatomy, growth, and disease of several joints of the human body, including the temporomandibular joint [3].

Animal studies have suggested that there is temporal bone and condylar adaptation in response to protrusive mandibular function. Shen and Darendeliler documented that the adaptive remodeling of the condylar cartilage proceeds with the biomolecular pathway initiating from chondrogenesis followed by osteogenesis. The theory of growth relativity essentially states that bone growth modification occurs relative to two elements: the retrodiscal tissues are stretched reciprocally, similar to a large elastic band, between the fossa and the displaced condyle during the expansion of the growing facial complex; and the transduction of these non-muscular forces has been shown to be effective at a significant distance from the actual physical soft tissue attachments [4].

Various techniques have been used to view the TMJ prior to the advent of MRI, which include lateral cephalograms, orthopantomograms and tomograms, bone scintigraphy with radiologic markers like 99mTc-MDP, arthroscopy, arthrography, and CT scanning. MRI, which is essentially a multiplanar imaging technique, is advantageous, owing to its ability to provide an accurate assessment of bony and soft tissues. Currently, MRI is opted for as the imaging modality of choice for assessing internal derangements of the temporomandibular joint. Ultrasonography (USG) is a highly reproducible method to evaluate the mandibular condyle cartilage, which is an economical and cost-effective diagnostic imaging method and could help study changes in the condylar cartilages [5].

On thorough examination of the currently available literature, functional changes, including bone remodeling and joint adaptations, have been studied using X-rays, histologic studies, electromyographic studies (EMG), ultrasound imaging, MRI, and computed tomography. In a study by TG Williams et al., bone appearance and shape were used to assess changes in cartilage thickness over time for individuals suffering from osteoarthritis (OA) [6].

Schmitz RJ et al. undertook a study to better understand how well clinical ultrasound thickness measures are associated with gold standard MRI measures by analyzing the femoral condylar cartilage thickness in OA patients. They concluded that there was a moderate to strong correlation between the MRI and USG measure of cartilage thickness in the medial femoral condyle [7].

However, no studies quantified the condylar cartilage activity in humans using MRI and USG. There is a need to develop easily accessible and cost-effective clinical tools to assess the changes in articular cartilage thickness.

Thus, the aim of this study is to evaluate the condylar cartilage thickness by using MRI and ultrasound imaging in patients undergoing myofunctional appliance therapy for mandibular advancement and to compare between MRI and USG.

## Materials and methods

A prospective evaluation involving convenience sampling of 10 participants was undertaken. The sample size was decided by using the formula:

n = N*X / (X + N - 1), where X = Zα/22 ­*p*(1-p) / (MOE)2.

The inclusion criteria involved growing patients exhibiting skeletal Class-II malocclusion with retrognathic mandible, patients exhibiting growth status in between CVMI II and III, patients undergoing myofunctional appliance therapy using a twin-block appliance, patients exhibiting a normodivergent to hypodivergent growth pattern. The exclusion criteria involved non-growing patients and patients exhibiting a growth status of CVMI IV. The diagnosis of participants who were given a twin block was based on clinical and radiological findings. The duration of the study was 18 months. For all patients, construction bite with horizontal advancement ranging between 5 mm and 6 mm and vertical advancement within freeway space ranging between 3 mm and 4 mm was obtained. All patients underwent MRI and ultrasound imaging to evaluate condylar cartilage thickness. The dimensional changes of the width of the condyle at the beginning (T0) and after six months of twin-block therapy (T1) were calculated by a single examiner who was a highly trained radiologist. Intraobserver reliability was 0.93. The thickness of condylar cartilage was measured in the area where maximum proliferative changes were seen. The USG and MRI were taken at some point of time but the operator was blinded for the measurement on MRI since it was measured at a different point of time, whereas the operator performed USG measurements chairside at the same point of time; this was done to avoid bias between USG and MRI readings. The final treatment results were not included in the study, as we wanted to primarily assess the changes in condylar cartilage thickness using different imaging modalities.

**Figure 1:**
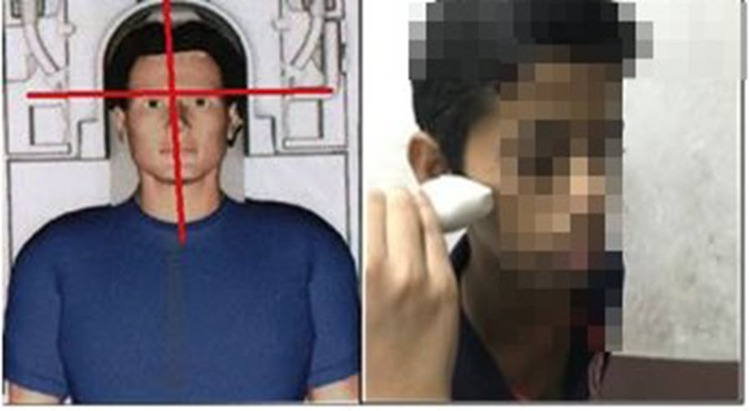
Patient position for MRI and ultrasound imaging

**Figure 2:**
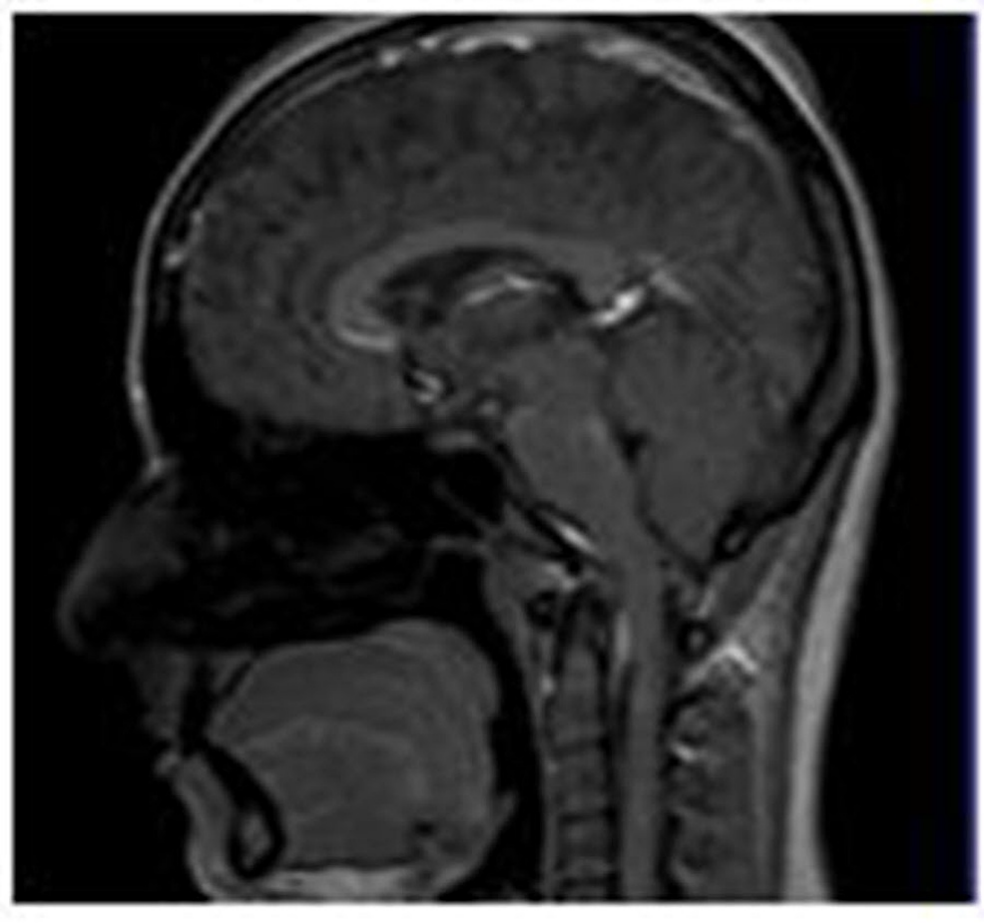
Sagittal view of TMJ using the MRI imaging modality TMJ: temporomandibular joint

The MRI scanner used was the Siemens 1.5 Tesla (Siemens AG, Munich, Germany) utilizing both the surface and brain coils. As part of the protocol, thin coronal and sagittal sections of the temporomandibular joint were acquired in both the closed and open mouth positions in two separate sessions labeled T0 and T1. The ultrasound scanner used for the study was the Hitachi-Aloka system (ProSound Alpha-6; Hitachi, Ltd., Tokyo, Japan) with a linear transducer working within the 7-12Mhz frequency bandwidth. Patient position for MRI and ultrasound imaging is illustrated in Figure 1. The sagittal view of the temporomandibular joint using the MRI imaging modality is shown in Figure 2.

**Figure 3:**
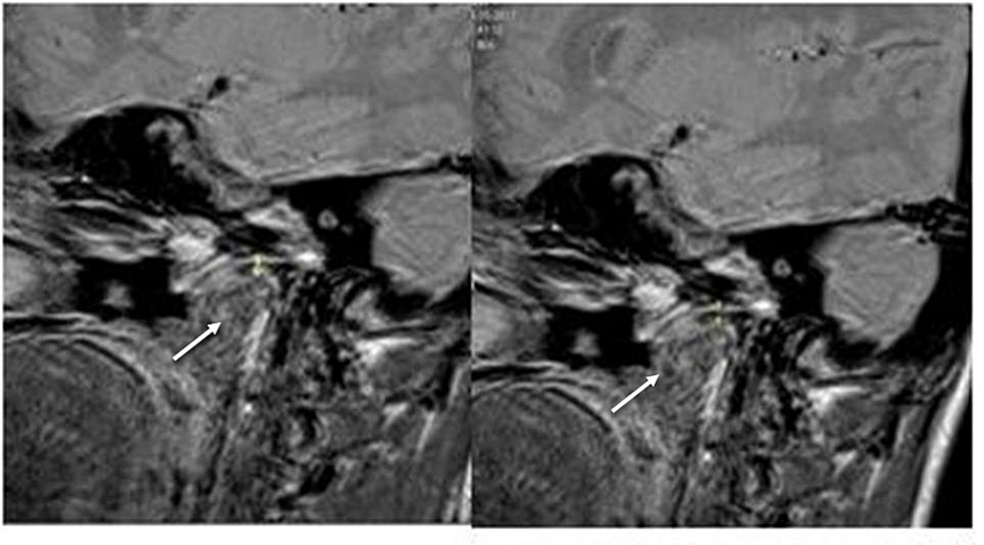
MRI images taken at T0 (left) and T1 (right) to demonstrate the dimensional changes of the condylar cartilage after twin-block therapy

**Figure 4:**
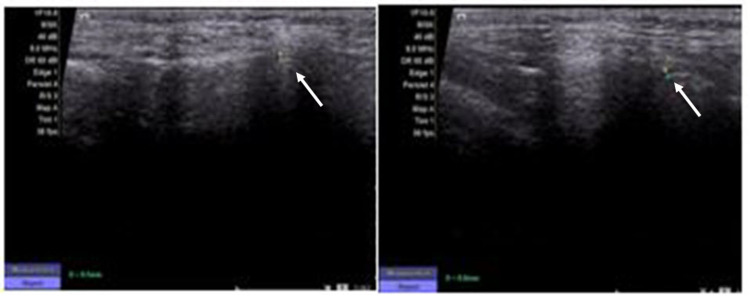
Ultrasound images taken at T0 (left) and T1 (right) to demonstrate the dimensional changes of the condylar cartilage after twin-block therapy

A comparative evaluation was done of all the data obtained from the MRI images and ultrasound imaging at T0 and T1 to appraise any dimensional changes in the thickness of the condylar cartilage. Figures 3-4 demonstrate the dimensional changes of the condylar cartilage, as evaluated by MRI and ultrasound imaging, respectively.

Data were analyzed by a between-group comparison using the unpaired t-test and a within-group comparison using the paired t-test.

## Results

There were statistically significant changes in p-value at T0 and T1 in the open and closed mouth position, on the left and right sides, as evaluated on MRI. The mean and standard deviations, t-test, and p-value of MRI at T0 and T1 in the open and closed mouth positions on each side are depicted in Table 1.

**Table 1:** Data obtained from MRIs in the open and closed mouth positions on the left and right sides at T0 and T1

Sr. No	Parameter	Mean in mm	N	Std. Deviation	Mean difference	t value	P-value
1	T0 (MRI), open mouth left side	0.49	10	0.0876	-0.28	-9.635	<0.001
	T1 (MRI) open mouth, left side	0.77	10	0.1252			
2	T0 (MRI)closed mouth, left side	0.467	9	0.0866	-0.311	-11.94	<0.001
	T1 (MRI) closed mouth, left side	0.78	9	0.044			
3	T0 (MRI), open mouth right side	0.97	10	1.4174	0.16	0.356	0.73
	T1 (MRI) open mouth, right side	0.81	10	0.0994			
4	T0 (MRI), closed mouth, right side	0.52	10	0.1033	-0.27	-12.65	<0.001
	TI (MRI) closed mouth, right side	0.79	10	0.0738			

**Figure 5:**
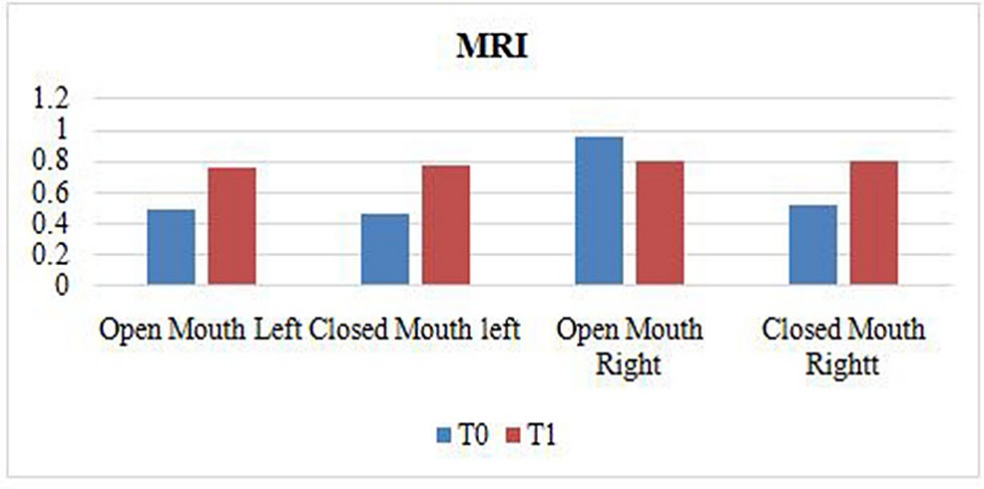
Graphical representation of data obtained from MRI images in the open and closed mouth positions on left and right sides at T0 and T1

At T0, the mean thickness noted was 0.49 and 0.48 during opening and closing on the left side and 0.52 in both positions on the right side. At T1, the mean thickness noted was 0.8 and 0.79 during opening and closing on the left side and 0.81 in both positions on the right side. This is graphically represented in Figure 5.

Similarly, ultrasound imaging showed consistent findings that were statistically significant. The mean, standard deviations, t-test, and p-value of ultrasound imaging at T0 and T1 in the open and closed mouth positions on each side are depicted in Table 2.

**Table 2:** Data obtained from ultrasound imaging in open and closed mouth position on left and right sides at T0 and T1

Sr no	Parameter	Mean in mm	N	Std. Deviation	Mean diff	t-value	P-value
1	T0 (USG), open mouth left side	0.49	10	0.0876	-0.31	-13.286	<0.001
2	T1 (USG) open mouth, left side	0.8	10	0.0816			
3	T0 (USG)closed mouth, left side	0.48	10	0.0919	-0.31	-13.286	<0.001
4	T1 (USG) closed mouth, left side	0.79	10	0.057			
5	T0 (USG), open mouth right side	0.52	10	0.0632	-0.29	-12.429	<0.001
6	T1 (USG) open mouth, right side	0.81	10	0.0994			
7	T0 (USG), closed mouth, right side	0.52	10	0.1033	-0.29	-9.222	<0.001
8	TI (USG) closed mouth, right side	0.81	10	0.0316			

**Figure 6:**
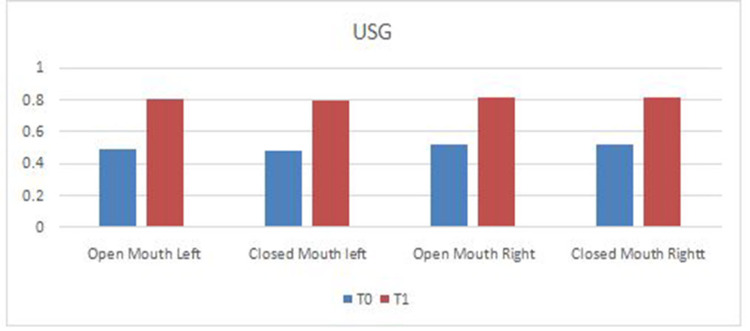
Graphical representation of data obtained from ultrasound images in the open and closed mouth positions on the left and right sides at T0 and T1

At T0, the mean thickness noted was 0.49 and 0.48 during opening and closing on the left side and 0.52 in both positions on the right side. At TI, the mean thickness noted was 0.8 and 0.79 during opening and closing on the left side and 0.81 in both positions on the right side. This is graphically represented in Figure 6.

**Figure 7:**
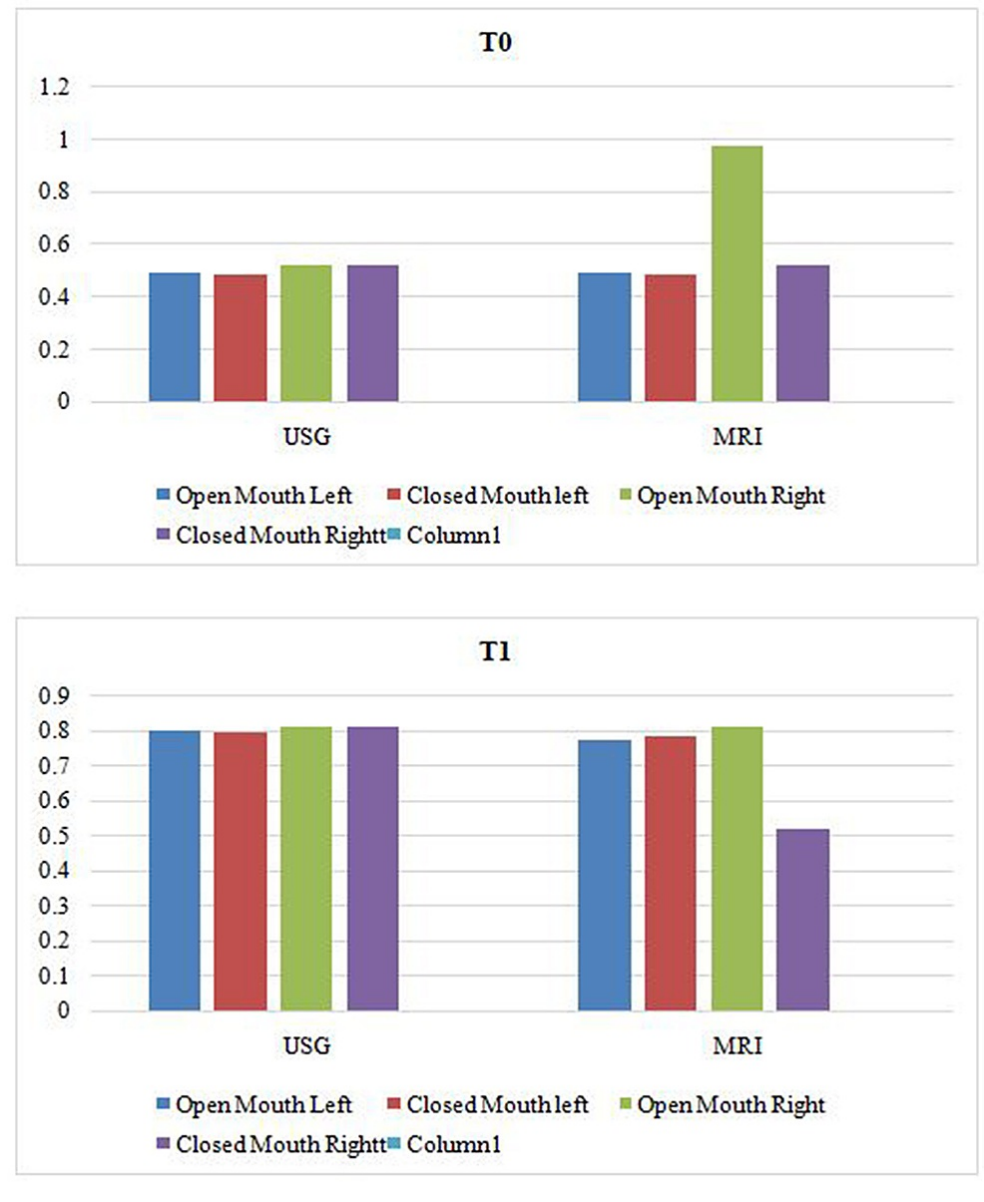
Comparative evaluation of data interpreted by MRI and ultrasound imaging at T0 and T1

There were no significant differences between the mean thickness of condylar cartilage as interpreted by MRI and ultrasound imaging at T0 and T1. It can be inferred that both imaging modalities are of diagnostic equality, as shown in Figure 7.

## Discussion

The alterations within the condyle-glenoid fossa complex and the positional changes of the glenoid fossa in the cranium after removable functional appliance therapy and after the completion of fixed appliance therapy can likewise be noted utilizing MRI. The MRI system is known to be non-intrusive and radiation-free and gives more prevalent difference determination than some other imaging modality [3]. However, it is endowed with several disadvantages such as increased cost and taking longer to procure, thereby minimizing patient comfort. Images are subject to unique artifacts that must be recognized and abated. It is non-compliant for patients with metal implants and foreign bodies, and careful attention to safety measures is necessary to avoid serious injury to patients and staff. Also, it requires special MRI-compatible equipment and stringent adherence to safety protocols. Therefore, we used ultrasound imaging in conjunction with MRI to obtain an insight into the dynamics of the TMJ.

High-resolution USG is an accurate, inexpensive, readily accepted by patients, and non-invasive method for imaging the musculoskeletal system. Articular cartilage can be visualized directly by USG at different peripheral joints, including the knee, elbow, wrist, shoulder, etc. It appears as a homogeneous anechoic band because of the high water content between the bony cortex and the soft tissues delimited by two sharp and hyper-echoic interfaces. The lack of echoes and the sharpness of the synovial space-cartilage and cartilage-bone inter-faces are the principal features of articular cartilage in healthy subjects [8-12].

The temporomandibular joint can be visualized using the closed, partially open, and open-mouth positions. MRI is a dependable procedure for evaluating the TMJ when evaluating its normalcy in the closed- and open mouth positions [13]. Over the years, imaging modalities like MRI and USG imaging have been readily used for determining the condylar position, disc displacement, glenoid fossa remodeling, and change in mandibular position. However, our study employs MRI and ultrasound imaging for evaluating the condylar cartilage thickness, owing to the effectiveness of the imaging systems and minimized radiation exposure.

Our study designates MRI as the gold standard for examination of the TMJ area for differentiating between soft tissues, evaluating dimensional changes of condylar cartilage. Ultrasound imaging, on the other hand, is a form of mechanical vitality that is transmitted through and into biological tissues as an acoustic pressure wave at frequencies over the farthest point of human hearing. Ultrasound imaging has been used to evaluate the condylar motion, to assess the morphology of masseter muscle detection of condylar involvement in children with juvenile idiopathic arthritis, TMJ disc displacements, and for glenoid fossa remodeling [12]. However, there is a lack of literature evaluating the changes in the thickness of the condylar cartilage after functional appliance therapy using these imaging modalities, necessitating the need for this study.

In 2003, Voudouris et al. attempted to improve on previous studies by combining several methods, including permanent implantation of electromyography (EMG) electrodes, computerized histomorphometry of bone formation, etc. They concluded that the glenoid fossa grows histologically in a downward and backward direction in control of nonhuman primates (similar to growth in humans) [4].

In 1997, Rabie et al. studied osteogenesis in the glenoid fossa in response to mandibular advancement at various time intervals in rats. The remodeling occurs by the expression of endogenous regulatory factors of cells in the mandibular condyle through an endochondral ossification process and intramembranous ossification in the GF [13-14].

In 2014, Santosh et al. evaluated and compared temporomandibular joint changes and the disk-condyle-fossa relationship following functional appliance therapy using twin-block and Bionator appliances using MRI. This study confirmed that the condyles occupied a more anterior position in the fossa to its pretreatment position while the disk moved more posteriorly in relation to the condyle; these findings are consistent with the findings in our study [1]. Mandibular protrusion leads to an increment in the number of replicating mesenchymal cells in the temporomandibular joint space. Individual variations in the response to growth modification therapy could be a result of the close interrelation between mesenchymal cell numbers and their growth [15-16].

There were moderate to strong correlations between MRI and USG measures of cartilage thickness in the medial femoral condyle in a study done by Schmitz et al. Even though USG may underestimate cartilage thickness relative to MRI in a single sitting, it is actually a useful clinical tool to assess cartilage thickness when serial investigations are performed [7].

In this study, both MRI and ultrasound imaging portrayed consistent findings showing statistically significant changes in p-value at T0 and T1 in the open and closed mouth positions, on the left and right sides. An additional observation noted in this study was that the condylar cartilage thickness of males was more as compared with females of the same age. However, these observations noted were beyond the scope of this study and further research is required to substantiate it. USG is viable and effective in measuring the outer marginal thickness of condylar cartilage, whereas MRI is effective in measuring the entire cartilage thickness. On the contrary, USG is more dynamic to measure thickness in the open and close mouth technique as compared to MRI because of the inherent cumbersome requirements for MRI, whereas USG is a simple chairside and cost-effective procedure. While MRI by its superior resolution of detail, penetration, and depiction of even deeper tissues, is the gold standard for assessing condylar cartilage thickness, the role of USG as a highly valuable diagnostic tool to assess condylar cartilage proliferation, especially when done serially cannot be understated, something which is required to assess the effectiveness of a given mandibular advancement appliance.

## Conclusions

Within the given consensus of this study, we concluded that the condylar cartilage thickness increases significantly after twin-block therapy suggestive of mandibular growth in skeletal Class-II Malocclusion. It can be inferred that both MRI and ultrasound imaging carry equal diagnostic interpretation, as there was no statistically significant difference between the two imaging modalities.
